# The relationship between parental neglect and cyberbullying perpetration among Chinese adolescent: The sequential role of cyberbullying victimization and internet gaming disorder

**DOI:** 10.3389/fpubh.2023.1128123

**Published:** 2023-03-23

**Authors:** Xiong Gan, Ke-Nan Qin, Guo-Xing Xiang, Xin Jin

**Affiliations:** Department of Psychology, College of Education and Sports Sciences, Yangtze University, Jingzhou, China

**Keywords:** parental neglect, cyberbullying perpetration, cyberbullying victimization, internet gaming disorder, adolescent

## Abstract

Cyberbullying perpetration and victimization have been common public health issues that have impaired the development of adolescent physical and mental health. Abundant research has proven associations between negative parental factors and cyberbullying perpetration. However, there is a paucity of research exploring the impact of parental neglect on cyberbullying and its internal mechanisms. Based on the parental acceptance-rejection theory (PARTheory) and the general aggression model (GAM), the present study constructs a sequential mediation model in which parental neglect is related to adolescent cyberbullying perpetration through cyberbullying victimization and internet gaming disorder (IGD). Using random cluster sampling, a total of 699 middle school students (*M*_age_ = 14.18 years, SD = 1.22, and 324 boys) were recruited from five schools in three provinces on mainland China. The participants completed questionnaires regarding parental neglect, cyberbullying perpetration, cyberbullying victimization, and IGD. The results of structural equation modeling indicated that parental neglect was positively associated with cyberbullying perpetration. The mediating effects of cyberbullying victimization and IGD in this relationship are significant both individually and jointly. The current findings have important implications for enlightening families and schools to pay particular attention to adolescents' experiences of parental neglect and provide them with timely feedback and assistance. This will contribute to the prevention and reduction of adolescent involvement in cyberbullying perpetration.

## Introduction

With the rapid advances in electronic technology, cyberbullying perpetration and victimization have become common public health problems among adolescent populations around the world (1). Regarding definition, cyberbullying refers to the intentional and repeated infliction of harm through the medium of electronic text (2), which peaks in adolescence (3). A number of researchers explored the prevalence of cyberbullying in different cultural contexts. For example, a study found that 10.0% of German adolescents reported cyberbullying others (4). Teenagers reported a cyberbullying victimization rate of 15.8% in the United States (5). In addition, a scoping review covering nine countries on youth cyberbullying showed that, in terms of victims, the prevalence median (23.0%) found in studies of China was remarkably higher than in other countries, such as Australia (5.0%), Sweden (5.2%), and Germany (6.3%) (6), meaning that this phenomenon was more serious in China. Recently, Rao et al. (7) conducted a study on cyberbullying based on 2,590 Chinese middle school students and revealed that 28.0% of these were perpetrators, 44.5% were victims, and 25.2% were both perpetrators and victims in the past 6 months. Besides, adolescence is a critical period for all aspects of an individual's development. Cross-cultural research found that suffering from cyberbullying was not conducive to the healthy development of adolescent psychology and behavior. Existing studies have demonstrated that participation in cyberbullying is closely associated with various mental health-related negative consequences (8), including depression, anxiety (9), loneliness (10, 11), hopelessness (12), and even suicidal ideation (13). Engaging in cyberbullying also causes several maladaptive developments in teenagers, such as disordered eating behaviors (14), more learning and school problems (15), violent and delinquent behaviors (16), problematic internet use (17), substance abuse, and suicide attempts (18). Given the high prevalence and adverse effects of cyberbullying among Chinese youth, examining the key factors linked to cyberbullying may be helpful in developing scientific interventions and protecting them from cyberbullying.

From a developmental perspective, the factors within the family system have the most direct and lasting effect on the individual's development (19). To our knowledge, previous studies have focused on the influence of family factors (e.g., parental variables) on adolescent cyberbullying (20), such as parenting styles, parental psychological control, and parental phubbing (21–23). However, few studies have explored the direct effect of parental neglect on cyberbullying perpetration and its internal mechanisms (24). In view of this, the current study will examine the relationship between parental neglect and cyberbullying perpetration as well as the roles of cyberbullying victimization and internet gaming disorder (IGD) in this association, which contributes to preventing and reducing youth cyberbullying from a family systems perspective.

### Parental neglect and cyberbullying perpetration

Neglect is the most common form of child maltreatment, yet it receives the least scientific and public attention (25). A meta-analytic review indicated that the global prevalence of physical and emotional neglect was 16.3 and 18.4%, respectively (26). Li et al. (27) conducted a survey of a sample of Chinese adolescents and found that the incidence of emotional and physical neglect was as high as 49.48 and 68.64%, respectively. Parental neglect is defined as parental actions that lack sufficient attention and responsibility for the children's basic needs or a failure to protect them from real or potential harm, which can manifest in physical, emotional, educational, and supervisory aspects (28–30). The parental acceptance-rejection theory (PARTheory) suggests that individuals who were rejected by their parents are more likely to report having mental health and behavioral problems than those who were accepted (31). It has been revealed that parental neglect is significantly correlated with psychological maladjustment and negative personality dispositions (e.g., hostility, negative self-esteem, emotional instability) (32). Previous literature has also shown that parental neglect leads to severe developmental outcomes in youth, including low academic competence, bad school adjustment (33), obesity (34), and violence (35). In addition, Van der Kolk (36) found that frustrating or traumatic experiences are extremely harmful to individual psychophysiological development (e.g., the hypothalamic-pituitary-adrenal axis, the amygdala, and the hippocampus).

According to the general aggression model (GAM), continued exposure to situations of parental neglect causes unpleasant feelings for adolescents, and the frustrating experiences further trigger aggressive tendencies and perpetrate aggressive behaviors (37). However, frustration did not necessarily lead to individuals' overt aggression due to social norms, namely, the expectation of punishment for aggressive behavior affects target selection (38). In other words, when confronted with parental authority, adolescents experiencing neglect do not often react excessively (e.g., by being hostile and fighting back), so they tend to choose more covert ways to cope with their negative emotions, like shifting their aggression from reality to online (12). An exploratory qualitative study also found that a crucial motivation for cyberbullies was to release their negative feelings (39). In addition, the social information processing (SIP) model (40) states that social behavior is a function of individuals' series of social information processing steps, which assumes that biased or flawed processing leads to deviant social behavior (e.g., aggression). Thus, adolescents who were neglected by their parents are more likely to access abnormal patterns of processing social information (41), such as increased hostile attribution bias, which in turn leads to cyberbullying perpetration (42). Extensive cross-country research has revealed that adolescents who have experienced parental neglect are more likely to engage in violent and aggressive behavior (43–45). Among Chinese teenagers, cross-sectional evidence showed that childhood physical and emotional neglect were positively associated with aggression (46, 47). Similarly, two longitudinal studies have suggested that physical and emotional neglect from parents effectively contributes to the increase in youth bullying and cyberbullying perpetration in China (48, 49). Furthermore, Wang and Jiang (24) have found that parental neglect plays a positive role in Chinese adolescent cyberbullying perpetration. Thus, parental neglect as a family risk factor may be linked to youth involvement in cyberbullying. Given the above theories and empirical studies, the current study proposes that parental neglect will have a positive relationship with cyberbullying perpetration among adolescents.

### The mediating role of cyberbullying victimization

As shown above, parental neglect has a serious impact on cyberbullying perpetration in youth (24). Some scholars have developed a view of this phenomenon and call it the “cycle of violence,” referring to the fact that previous experiences of neglect increase the risk of individuals engaging in violent behaviors later on (41, 50). However, it should be noted that Widom (51) subsequently described a new finding that the experience of maltreatment (including neglect) also increases the likelihood of individual revictimization, known as the “cycle of victimization.” According to the perspective of “target vulnerability” (52), certain personal characteristics (e.g., psychological distress) put adolescents at risk by “fitting” into the needs, motivations, and responses of potential perpetrators. In particular, individuals maltreated in childhood tend to be withdrawn, fearful, tense, worthless, and unloved (53). Then, these attributes disrupt their coping style and ability to resist victimization, ultimately making them more likely to be targeted by perpetrators online (54). According to the “cycle of victimization,” parental neglect may be linked to cyberbullying victimization. Empirical studies have provided a wealth of evidence to support the protective role of positive parental behaviors in youth cyberbullying victimization (55–57). Favorable parent-child relationships (e.g., positive parent-child communication) have been found to be effective in preventing young people from suffering from cyberbullying victimization (58). By contrast, a systematic literature review examining the impact of family factors on cyberbullying found that young people with poor relationships with their parents (suffering from neglect) are more likely to be cyberbullying victims (20). In addition, a study of 1,025 Chinese adolescents has indicated that childhood maltreatment, including physical neglect and emotional neglect, plays a positive role in their cyberbullying victimization (59). As a result, this study speculates that parental neglect would also relate to adolescent cyberbullying victimization positively.

Furthermore, it has been shown that cyberbullying victimization acts as the strongest predictor of cyberbullying among adolescents (60). Similarly, according to the GAM, the experience of online victimization evokes bad moods (e.g., anger and frustration) in individuals, resulting in some form of aggression (37). The smaller the punishment expectation, the greater the likelihood that aggression will occur (38). The anonymity of cyberspace provides a relatively safe environment for the bullied to fight back (8), and the various aggressive cues that appear online are constantly perceived and reinforced by the bullied, causing them to be more inclined to turn to bullying others online (61). The relationship between cyberbullying victimization and perpetration has been extensively researched by many scholars. For example, Wong et al. (62) employed a sample of 1,917 secondary school students for their study in China and found that both participation in traditional bullying behaviors and experiencing cybervictimization were significantly associated with an increased propensity to cyberbully others. Shi et al. (63) and Wang et al. (64) provided cross-sectional evidence for the predictive effect of Chinese adolescent cyberbullying victimization on their cyberbullying perpetration. Two longitudinal studies on adolescents have also shown that early individual experiences of cybervictimization positively predicted their subsequent cyberbullying perpetration (65, 66). In other words, there is a high risk that cyber victims may turn into cyber bullies (1). Moreover, it has been suggested that cyberbullying victimization can play a mediating role between the parent-child relationship and externalizing problem behaviors (58). Inspired by the previous literature, the current study proposes that cyberbullying victimization will mediate the relationship between parental neglect and cyberbullying perpetration among adolescents.

### The mediating role of internet gaming disorder

IGD refers to an individual's uncontrollable, excessive, and compulsive use of online games that causes social and/or emotional problems (67). Extensive research has demonstrated that IGD has numerous negative developmental consequences for individuals in terms of functional brain structure (68), mental health (69), sleep problems (70), academic achievement (71), and other externalizing behaviors (72). Given the detrimental effects of IGD, it is urgent to explore its critical influences. As far as adolescent use of internet gaming is concerned, the role of the parents in the family should not be overlooked. A systematic review has indicated that poor parental behaviors and attitudes increase the risk of adolescent involvement in internet addiction (73). According to the social control theory (74), parent-child intimacy can inhibit problematic behaviors. However, it can also exacerbate problematic behaviors (e.g., IGD) due to the weakness of the emotional bond between neglected adolescents and their parents. The compensatory satisfaction theory also suggests that pathological internet use is the result of missing real psychological needs being compensated for online (75), meaning that neglected adolescents use online games to satisfy basic psychological needs and eventually indulge in IGD. Previous studies have provided empirical evidence of the predictive effect of poor parental behaviors on adolescent addictive behaviors (76). For instance, Kwak et al. (77) investigated a sample of 1,170 Korean adolescents and found that parental neglect predicted an increase in the probability of their smartphone addiction. A study on the relationship between child maltreatment and internet addiction points out that both psychological and physical neglect positively predict internet addiction among adolescents (78). In addition, Lin et al. (79) and Xie et al. (80) have revealed that middle school students with experiences of parental neglect report a high level of IGD in China. That is, there may also be a positive correlation between parental neglect and adolescent IGD.

Besides, a relationship between internet use disorder and cyberbullying perpetration has been found (81–83). The former acts as a persistent urge to connect to the internet, affecting the individual's mood (e.g., increasing depression, hostility, and social anxiety) (84), which in turn leads to violent or aggressive behaviors (85, 86). Frequent exposure to and use of the internet endangers individuals' safety and mental health and increases cyberbullying, including flaming, harassment, cyberstalking, and denigration (87). Previous studies have demonstrated that addictive behaviors (e.g., smartphone addiction and internet addiction) predict high levels of cyberbullying perpetration, whether among teenagers or university students (24, 88–90). A longitudinal study on the relationship between risky online behaviors has indicated that problematic internet use predicts an increase in subsequent cyberbullying perpetration among Chinese youth (91). Gan et al. (92) and Nwanosike et al. (93) also found that IGD had a positive impact on both traditional bullying and social bullying behaviors among adolescents and undergraduate students. Considering that IGD is also an internet use disorder, this study has reason to assume that IGD has a strong association with cyberbullying perpetration in youth. Moreover, previous research has also suggested the mediating effect of IGD between environmental variables and bullying (92). Addictive behaviors (e.g., smartphone addiction) can also exacerbate the effects of parental neglect on cyberbullying perpetration (24). Given the above theoretical and empirical evidence, the current study proposes that IGD will mediate the relationship between parental neglect and cyberbullying perpetration among adolescents.

### Cyberbullying victimization and internet gaming disorder

As mentioned above, both cyberbullying victimization and IGD play mediating roles between parental neglect and cyberbullying perpetration in teenagers. It is worth noting that previous theoretical and research evidence suggests that cyberbullying victimization and IGD are closely linked. According to the general strain theory (GST), as proposed by Agnew (94), the stimuli that individuals receive in real life that present negative values (e.g., cyberbullying victimization) can lead to their deviant behaviors. Furthermore, the social compensation theory states that online victimization embodies negative social interactions and that individuals compensate for interpersonal deficiencies by using the internet (95). Therefore, as a developmental problem, IGD could be affected by the experience of cyberbullying victimization. Prior research has explored the association between traditional and/or cyber victimization and addictive behaviors (96). For instance, ample evidence has found that peer victimization is a strong predictor of problematic online game use and IGD among Chinese teenagers (97–99). A study of 1,000 adolescents revealed that being involved in cyberbullying victimization had a positive effect on their problematic internet use (58). Moreover, Lin et al. (100) and Xin et al. (101) surveyed a large number of Chinese students and showed that their level of cyberbullying significantly predicted an increased risk of internet addiction. That is, it is reasonable to conclude that the level of cyberbullying victimization is positively related to the risk of young people engaging in IGD. In summary, the current study proposes that cyberbullying victimization and IGD will have a sequential mediation effect on the relationship between parental neglect and cyberbullying perpetration among adolescents.

### The current study

To sum up, previous studies have explored many parental variables of adolescent cyberbullying perpetration (22, 23, 102), but there is little focus on parental neglect. Meanwhile, despite some theories and perspectives suggesting a correlation between parental neglect and cyberbullying and the existence of psychosocial mechanisms, there has been a lack of sufficient empirical evidence to date. Grounded on the above-mentioned studies and theories, the aim of this study is to address the research gaps by investigating the relationship between parental neglect and cyberbullying perpetration and constructing a sequential mediation model with the following hypotheses: (1) parental neglect will have a positive relationship with cyberbullying perpetration; (2) cyberbullying victimization will mediate the relationship between parental neglect and cyberbullying perpetration; (3) IGD will mediate the relationship between parental neglect and cyberbullying perpetration; and (4) cyberbullying victimization and IGD will have a sequential mediation effect on the relationship between parental neglect and cyberbullying perpetration.

## Method

### Participants and procedures

The participants in this study were recruited from five public middle schools in Hubei, Shaanxi, and Sichuan provinces on mainland China by random cluster sampling. A total of 699 adolescents ranged in age from 12 to 17 years (*M*_age_ = 14.18 years, SD = 1.22), of which 324 were boys (46.4%). The current study was approved by the Research Ethnics Committee of the College of Education and Sports Sciences, Yangtze University. Prior to starting the formal data collection, informed consent was obtained from the school leaders and students for this study. Adolescents were informed of several important research principles, including anonymity, independence, non-harmfulness, and voluntariness, namely the guarantee of confidentiality of information about participants and their right to withdraw from the survey at any time. The whole procedure was carried out by well-trained teachers and research assistants during school time. All students were encouraged to be honest and required to complete a paper-and-pencil questionnaire regarding demographic information and study measurement tools in ~20 min on a class basis. In addition, participants in this study did not receive any form of gift in return.

### Measures

#### Cyberbullying victimization/perpetration

This study uses the Cyber Victimization/Bullying Scale to measure cyberbullying victimization and cyberbullying perpetration among adolescents in the last 7 days (103). The scale contains 12 items, of which the first six are used to assess cyber victimization (e.g., “Some people have laughed at me *via* email, mobile phone text messages, online instant messaging (QQ, WeChat), and social networking sites (Qzone, Renren, WeChat Moments)”) and the second six to assess cyber bullying [e.g., “I have threatened people *via* email, mobile phone text messages, online instant messaging (QQ, WeChat), and social networking sites (Qzone, Renren, WeChat Moments)”]. All items were rated on a 7-point Likert scale from 0 (never) to 6 (six times or more). The mean scores were calculated, with higher scores meaning a higher degree of cyber victimization or bullying. This scale has demonstrated good reliability and validity among adolescents (104, 105). In the current study, the Cronbach's alpha coefficient for the whole scale was 0.92. The Cronbach's alpha coefficients for the cyber victimization and cyber bullying subscales were 0.91 and 0.93, respectively.

#### Parental neglect

This study used the neglect subscale of the Child Psychological Abuse and Neglect Scale to evaluate the condition of adolescent parental neglect (29). The 17-item subscale is divided into three dimensions, including emotional neglect (e.g., “My parents do not comfort me when I am sad or scared”), educational neglect (e.g., “My parents do not care about changes in my academic performance”), and physical and supervisory neglect (e.g., “My parents forbade me to play cards and gamble”). Participants rated the items on a 5-point Likert scale from 1 (none) to 5 (always). After reversing the scoring of some items, the mean scores were calculated, with the higher scores representing the higher levels of parental neglect. The subscale had good reliability and validity in adolescents in China (79, 80). In the current study, the Cronbach's alpha coefficient was 0.92.

#### Internet gaming disorder

This study used the Internet Gaming Disorder Questionnaire to assess the frequency of IGD symptoms among adolescents over the past 6 months (106, 107). The questionnaire consists of 11 items (e.g., “Have you ever needed extra money from friends or family because you spent too much money on video game devices, software, or games/internet”), which were rated on a 3-point Likert scale from 0 (never) to 2 (frequently). Then the scores of all items were recoded as: 0 = “never,” 0.5 = “sometimes,” and 1 = “frequently.” This scoring method could take into account participants who occasionally experienced IGD symptoms and increase the accuracy of the measure (107). The mean scores were calculated, with higher scores reflecting a higher risk of IGD. Previous studies have demonstrated that this questionnaire has good reliability and validity among Chinese adolescent samples (92, 108). In the current study, the Cronbach's alpha coefficient was 0.83.

### Statistical analyses

SPSS 25.0 and MPLUS 8.3 were used for data analysis in this study. First, we conducted Harman's single-factor test to examine common method biases in the collected data. Second, we performed descriptive statistics and correlation analyses for the main variables with SPSS 25.0. Third, we tested the sequential mediation model through structural equation modeling (SEM) with latent variables by using MPLUS 8.3 (109). Specifically, the process of testing the model was divided into three steps: in the first step, we examined the measurement model using confirmatory factor analysis (CFA); in the second step, we constructed the SEM from the independent variables to the dependent variable; and in the third step, we constructed the final SEM after incorporating the mediating variables on the basis of the above. The maximum likelihood estimation method was used to estimate and test all the models mentioned above. According to previous studies, we adopted the following criteria to evaluate whether the model fit was good: χ^2^*/df* < 5, *CFI* and *TLI* > 0.9, and *RMSEA* and *SRMR* < 0.08 (110, 111). This study conducted the bias-corrected percentile bootstrap method with 5,000 replicates to test the indirect effects of the hypothesis model. The 95% CIs without a zero indicated that mediating effects were statistically significant (112). Furthermore, several researchers have found that sex and age are the important factors in parental neglect, IGD, and cyberbullying, which factors are often used as control variables (80, 92, 98). Hence, sex and age were controlled for in the subsequent data analyses of this study.

## Results

### Preliminary analyses

Because the data was derived from the subjects' self-reported results, there is a possibility of common method bias (113). The current study used anonymization and reverse scoring to control for common method biases when distributing questionnaires (114). In addition, this study used two methods to examine the data for common method bias. First, the results of Harman's single-factor test showed that there were 8 factors with a characteristic root > 1, and the interpretation rate of the first factor was 26.93%, <the 40% critical standard, indicating that the common method bias of our study is not serious. However, some scholars have suggested that the single-factor test is problematic and recommend that the method controlling for the effects of a single unmeasured latent factor should be selected for testing common method bias (115, 116). In view of the above, this study first constructed the CFA model (M1) and then constructed another model (M2) by adding the method factor based on M1. A comparison of the main fit indexes for M1 and M2 showed that: Δ*TLI* = 0.02, Δ*CFI*= 0.03, Δ*RMSEA*= 0.004, and Δ*SRMR*= 0.01. The changes in fit indices were all <0.05, indicating that the model was not significantly improved by the inclusion of the method factor (117, 118). Therefore, both methods proved that there was no significant common method bias in this study.

The results of descriptive statistics and correlation analyses are shown in Table 1. As can be seen in the table, parental neglect, cyberbullying victimization, IGD, and cyberbullying perpetration were all significantly and positively correlated with each other (*ps* < 0.001). The correlation coefficients ranged from 0.25 to 0.56.

**Table 1:** Descriptive statistics and bivariate correlations of key variables.

**Variables**	**Descriptive statistics**				**Correlation coefficients (** ***r*** **)**			
	***M***	***SD***	***Skewness***	***Kurtosis***	**1**	**2**	**3**	**4**
1. PN	32.64	13.60	0.79	0.09	1.00			
2. CV	2.90	6.45	2.86	8.45	0.32^***^	1.00		
3. IGD	14.34	3.59	1.12	0.66	0.25^***^	0.31^***^	1.00	
4. CP	1.12	4.04	4.57	22.35	0.28^***^	0.56^***^	0.36^***^	1.00

PN, Parental Neglect; CV, Cyberbullying Victimization; IGD, Internet Gaming Disorder; CP, Cyberbullying Perpetration. ***p < 0.001.

### Serial mediation effect analyses

To explore the mediation effects of cyberbullying victimization and IGD, the current study constructed a sequential mediation model that consisted of four latent variables: parental neglect, cyberbullying victimization, IGD, and cyberbullying perpetration. Specifically, parental neglect includes the three observed variables of emotional neglect, educational neglect, and physical and supervisory neglect. Considering that the measures of cyberbullying victimization, IGD, and cyberbullying perpetration scales were unidimensional, all items on each of the above scales were divided into two, three, and two observed variables, respectively, by the item-structure balance method (119, 120), which was instrumental in maintaining the estimation stability of the model and improving the fit of the model. Then, this study used the CFA to test the measurement model, and the results revealed that χ^2^/*df* = 3.27, *CFI* = 0.98, *TLI* = 0.97, *RMSEA* = 0.06, and *SRMR* = 0.03, showing the good fit of the model.

**Figure 1:**
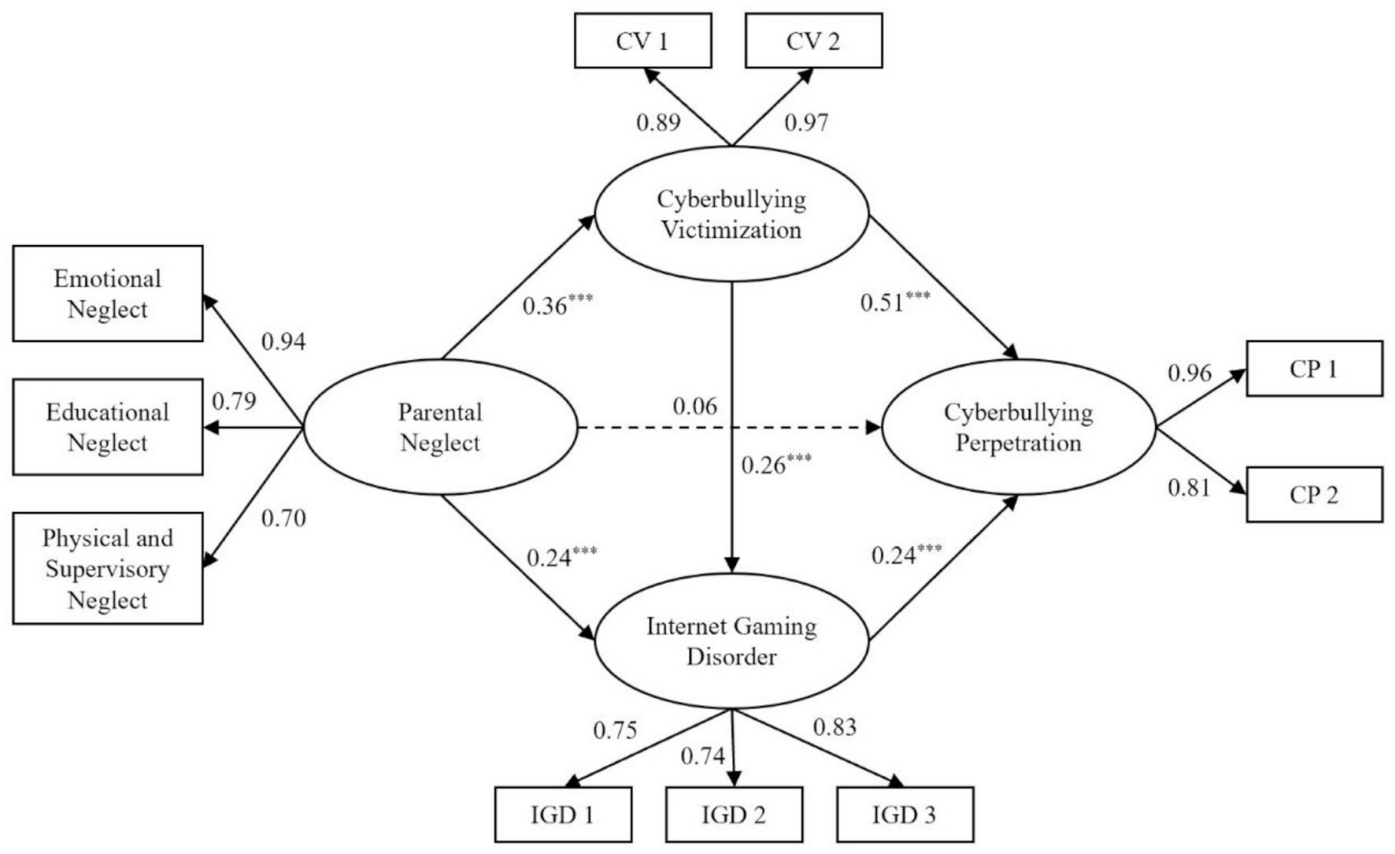
The effect of parental neglect on cyberbullying perpetration through cyberbullying victimization and internet gaming disorder. Control variables were omitted in the presentation. ^***^p < 0.001.

After controlling for sex and age, the current study conducted the sequential mediation model analyses by following several steps. First, the direct association between parental neglect and cyberbullying perpetration was tested. The result showed that χ^2^/*df* = 2.58, *CFI* = 0.99, *TLI* = 0.98, *RMSEA* = 0.05, and *SRMR* = 0.04, indicating a good fit to the data. Parental neglect was positively associated with increased cyberbullying perpetration (β = 0.33, *p* < 0.001). Then, we added two mediators, cyberbullying victimization and IGD, to the model. The results of the sequential mediation model showed that χ^2^*/df* = 3.84, *CFI* = 0.97, *TLI* = 0.95, *RMSEA* = 0.06, and *SRMR* = 0.04, indicating that the model fit well. As shown in Figure 1, parental neglect had a positive relationship with cyberbullying victimization (β = 0.36, *p* < 0.001) and IGD (β = 0.24, *p* < 0.001). Cyberbullying victimization was positively related to IGD (β = 0.26, *p* < 0.001) and cyberbullying perpetration (β = 0.51, *p* < 0.001). Moreover, IGD also was positively connected with cyberbullying perpetration (β = 0.24, *p* < 0.01). However, the direct relationship between parental neglect and cyberbullying perpetration was nonsignificant (β = 0.06, *p* = 0.18). The bootstrapping analysis was employed to test the sequential mediation effects. The findings, demonstrated in Table 2, revealed that the three mediation effects were all significant. Besides, the effect values of these three indirect pathways accounted for 58.62, 17.24, and 6.90% of the total effect, respectively.

**Table 2:** The sequential mediation effects of mediation model.

	**B**	**Boot SE**	**Boot 95%CI**
1. PN-CV-CP	0.17	0.04	[0.10, 0.27]
2. PN-IGD-CP	0.05	0.03	[0.02, 0.10]
3. PN-CV-IGD-CP	0.02	0.01	[0.01, 0.04]
4. Total indirect effect	0.24	0.05	[0.16, 0.34]
5. Total effect	0.29	0.06	[0.19, 0.41]

PN, Parental Neglect; CV, Cyberbullying Victimization; IGD, Internet Gaming Disorder; CP, Cyberbullying Perpetration. Bootstrap sample size = 5000. CI, confidence interval.

## Discussion

As cyberbullying has serious impacts on adolescent physical and mental health (1), researchers are devoted to exploring its related factors. Ample evidence exists indicating significant associations between negative family factors and adolescent cyberbullying perpetration (12, 23, 102). Nonetheless, there are few studies on the co-occurrence of parental neglect and cyberbullying perpetration, and the factors that can mediate the effect of the former on the latter have not been clarified. In view of this, this study aims to explore the association between parental neglect and cyberbullying perpetration and examine the roles of cyberbullying victimization and IGD behind this association in the Chinese context. Overall, the findings support the above hypotheses, which are discussed in detail in the following paragraphs.

### Parental neglect and cyberbullying perpetration

As expected, the first hypothesis about the direct relationship between parental neglect and cyberbullying perpetration was confirmed, consistent with the findings of prior research (24, 48). The result revealed a positive link between parental neglect and cyberbullying perpetration. This enriches the PARTheory and validates its applicability in explaining the link between parental neglect and cyberbullying perpetration (31). This current finding also provides cross-sectional evidence from Chinese adolescents for the GAM that parental neglect as a negative situational factor can have a negative impact on adolescents, which further enhances the risk of adolescents bullying others online (37). Besides, this result is also congruent with the SIP model (40), deriving from the fact that parental neglect experiences make adolescents process social information abnormally so as to increase their aggressive behavior online (41). A possible explanation of developmental traumatology for this finding is the description that parental neglect as a traumatic event affects the normal development of the individual's brain structure and function and then leads to the failure of self-regulating behaviors and acting out behaviors, such as cyberbullying perpetration (121). Furthermore, since our result only reflects the influence of the parenting role in the family system on adolescent cyberbullying, it is worth noting the contribution of child-related family factors, especially filial piety (42, 122), to the development of cyberbullying in Asian culture. As a result, this finding not only suggests that families and schools should try their best to provide a healthy growth environment for adolescents to reduce their adverse experiences, but also pay more attention to monitoring and investigating the status of cyberbullying regularly.

### The mediating role of cyberbullying victimization

The results of this study revealed that cyberbullying victimization could mediate the relationship between parental neglect and cyberbullying perpetration, in line with hypothesis 2. The findings reveal that parental neglect not only has a direct effect on cyberbullying perpetration but also has indirect effects through cyberbullying victimization. First, parental neglect was positively associated with cyberbullying victimization, consistent with the previous findings (59). This study also validates and extends the perspective of the “cycle of victimization” by showing that individuals who have been neglected tend to be more vulnerable to revictimization (51). Considering that one potential reason for this is the influence of social learning (123), the experience of long-term neglect in the parent-child relationship can lead individuals to believe that such victimized interactions in the network are also normal (54), thus weakening their ability to discover their victimization (54). Second, cyberbullying victimization was positively connected with cyberbullying perpetration, which is consistent with the prior studies (63, 64) and the viewpoint of the GAM (37). Due to the anonymity and diminished power differentials of the online environment, cyber-victims are more likely to have a greater chance of successfully fighting back or choosing to bully others through the internet compared to traditional victims (124–126). This approach to cyberbullying as a form of cyber revenge may represent a restoration of the balance of power and contribute to a greater sense of control and less powerlessness for the victim (127). In addition, it is noted by some researchers that the roles of perpetrators and victims in cyberbullying can easily shift into opposites (61). This study discussed and established the relationship between the two dimensions of cyberbullying separately, which facilitated a shared understanding of youth cyberbullying from the perspective of both the victim and the perpetrator to identify more targeted and efficient prevention and intervention strategies. This reveals to practitioners the need not only to help students build good relationships with their parents but also to work together with their parents to differentiate and help young people who play different roles in cyberbullying.

### The mediating role of internet gaming disorder

The present study found that IGD could also mediate the relationship between parental neglect and cyberbullying perpetration, verifying hypothesis 3. The findings are similar to previous studies (24, 92), that is, adolescents who suffer from parental neglect are more prone to IGD and are then motivated to attack or bully others through the internet. On the one hand, parental neglect was positively associated with IGD, in line with prior findings (79, 80), validating both the social control theory (74) and the compensatory satisfaction theory (75). One possible explanation for this is that prolonged exposure to a family that lacks parental care and supervision can lead to a lack of control and discipline in adolescents, causing a range of more problematic behaviors (74). Another explanation is that teenagers who have experienced neglect do not receive the basic need for family affection, and online gaming compensates for this lack, but excessive online gaming use causes IGD (75). On the other hand, IGD has a positive relationship with cyberbullying perpetration. The result is also similar to previous studies (17, 24, 90) that suggest that addiction to the internet (e.g., online games) or social media drives youth to perpetrate more cyberbullying. Excessive and uncontrolled use of online gaming acts as a negative environmental stimulus that provokes emotional and physical discomfort in individuals (67, 84), thus facilitating their cyberbullying perpetration (85–87). Besides, the problem-behavior theory suggests that adolescent problem behaviors are interrelated (128), which is consistent with the current research findings. At the same time, since the anonymity of the internet can help cyberbullies escape punishment more easily, they are likely to commit more acts of cyberbullying perpetration (8, 48). Therefore, teachers and parents also need to pay more attention to adolescent IGD behaviors and help them become aware of the links and dangers of IGD and cyberbullying in order to effectively prevent a vicious cycle of cyberbullying.

### Cyberbullying victimization and internet gaming disorder

The results of SEM indicated a positive association between cyberbullying victimization and IGD. The finding suggests that cyberbullying victimization and IGD play roles in the relationship between parental neglect and cyberbullying perpetration both individually and jointly, conforming to hypothesis 4. This reveals that the more experience young people have with cyberbullying victimization, the more likely they are to be addicted to online gaming, similar to the findings of prior research (58, 100, 101). The current findings provide support for the general applicability of GST in the field of cyberbullying and find that the negative values that victims of cyberbullying actually present to young people are an important factor in their involvement in IGD (94). Moreover, cyberbullies use online games as a coping strategy to escape emotional distress and interpersonal stress, and excessive use of internet games leads to IGD (96, 98), which is consistent with social compensation theory (95). Adolescents can effectively satisfy their psychological needs and regain self-esteem and confidence to mitigate and cope with the negative effects of peer victimization by immersing themselves in the world of online gaming (129). Accordingly, educators should focus on adolescent experiences of victimization online and be alert to the severity of their IGD to monitor them away from problematic behaviors and provide them with the necessary help and support in the first instance.

### Limitations and future directions

Although our study obtained relatively rich findings, there are still some limitations that need to be mentioned. To begin with, because our data was derived from subjects' self-reports, it may contain biases such as social desirability effects and memory bias. Future research should employ multiple measures (e.g., observation and/or follow-up methods) (59) and multiple sources to obtain self-reported data (e.g., parents, teachers, and peers). This would help to improve the reliability of the findings. Secondly, the cross-sectional design of the current study prevents us from inferring causal relationships between the research variables. Thus, subsequent studies could be conducted with some experimental interventions or longitudinal designs (91, 130) in order to determine the sequential effects and long-term impacts of parental neglect on adolescent cyberbullying perpetration. Thirdly, the results of this study were based on a limited sample of youth and were therefore less generalizable. In the future, researchers could include groups of different ages and cultures to test the cross-group and cross-cultural applicability of the framework model developed. Fourth, while our study develops a structural equation model of latent variables, it still does not consider whether different dimensions of parental neglect have different degrees of effect on other outcome variables. As a result, if future research could combine and discuss the separate and joint effects of each dimension, practitioners would be provided with more specific scientific guidance on parental neglect. Fifth, this study focused on the impact of negative parental behavior on problematic developmental outcomes in adolescents but lacked an exploration of the associated protective factors. Future studies should consider the effects of both risk and protective factors on adolescent development to gain a more integrated perspective on understanding their adaptive development. Finally, this study only discussed the influence of an environmental variable in the family system on adolescents' IGD and cyberbullying. Therefore, later studies could consider the impact of other systems on the adolescent development of problematic behaviors, such as schools and communities (88, 92). This would facilitate a more comprehensive perspective on the developmental antecedents of adolescents.

### Implications and recommendations

The current findings have significant implications for both theoretical research and practical application. With regard to theoretical contributions, first of all, this study contributes to the understanding of the relationship between parental neglect and adolescent cyberbullying perpetration and its internal mechanisms of cyberbullying victimization and IGD. These findings deepen previous research and extend the findings in the field of cyberbullying. Second, this study also uses a number of theories to argue for associations between key variables, facilitating the generalizability and explanatory power of these theories for relevant variables' relationships. Third, fewer studies have examined the relationship between parental neglect and cyberbullying perpetration from the perspective of the parental factors, for which this study provides empirical evidence from a group of adolescents in a Chinese cultural context.

In terms of practical implications, first, our study demonstrates a positive relationship between parental neglect and cyberbullying perpetration, suggesting that parents and teachers should pay particular attention to youth who have experienced parental neglect. The schools could conduct regular follow-up screenings to identify these young people. Teachers should work with parents to reduce the risk of cyberbullying perpetration for them. For example, interventions that increase parental companionship and enhance parent-child relationships should be provided (131). Second, we have found that cyberbullying victimization mediates the link between parental neglect and cyberbullying perpetration. In light of this, it is equally important to distinguish the role of youth in participating in cyberbullying. In particular, teachers and parents should encourage cyberbullying victims to report incidents so that students can receive immediate assistance and support to reduce the risk of being victimized and triggering cyberbullying (59). In addition, high levels of cyberbullying victimization have been found to be associated with high levels of IGD. Consequently, helping youth who are cyberbullied could also reduce the incidence of IGD. The schools could also provide the necessary counseling or group counseling for students engaged in cyberbullying. It has been shown that class-based short-term interventions have been effective in reducing media violence use and aggression among adolescents (130). Third, the results indicate that IGD also has a positive connection with cyberbullying perpetration. IGD is often used as a way for adolescents to compensate for basic psychological needs (75), implying that educators should focus on the establishment of parent-child and peer relationships and the appropriateness of internet use among adolescents. Specifically, regular mental health programs in schools could train and develop adolescents' social skills and ways to effectively control their internet use, both of which would help meet their basic psychological needs and decrease the development of non-adaptive internet use behaviors. Furthermore, researchers have proposed motivational interviewing as a promising approach based on students' motivation to change a problem behavior and then helping them to change it (132).

## Conclusion

Based on many theories, such as the PARTheory and the GAM, this study examines the relationship between parental neglect and cyberbullying perpetration and the mechanisms that mediate this relationship in Chinese youth. The current findings suggest that parental neglect is positively associated with cyberbullying perpetration. Moreover, cyberbullying victimization and IGD mediate the relationship between parental neglect and adolescent cyberbullying perpetration, both individually and jointly. These results suggest that educators should focus on adolescent experiences of parental neglect and online victimization, as well as the severity of IGD, for effective prevention and reduction of cyberbullying perpetration.

## Data availability statement

The raw data supporting the conclusions of this article will be made available by the authors, without undue reservation.

## Ethics statement

The studies involving human participants were reviewed and approved by the Research Ethics Committee of the College of Education and Sports Sciences, Yangtze University. Written informed consent to participate in this study was provided by the participants' legal guardian/next of kin.

## Author contributions

XG, K-NQ, and G-XX designed the study. XG collected data. K-NQ analyzed data and drafted the manuscript. G-XX reviewed and revised the manuscript. XJ participated in data collection. All authors contributed to the article and approved the submitted version.
